# Exploring patient-reported outcome measures to assess symptoms of moderately-to-severely active Crohn’s disease in adult and adolescent patients: a qualitative study

**DOI:** 10.1186/s41687-025-00959-1

**Published:** 2025-11-04

**Authors:** Theresa Hunter Gibble, Katherine Kosa, Bonita Basnyat, Susan Martin, Richard E. Moses, Payal Jha, Marla C. Dubinsky

**Affiliations:** 1 Eli Lilly and Company, Indianapolis, IN USA; 2 RTI Health Solutions, Research Triangle Park, Durham, NC USA; 3 RTI Health Solutions, Ann Arbor, MI USA; 4 Icahn School of Medicine at Mount Sinai, New York City, NY USA

**Keywords:** Crohn’s disease, Patient-reported outcomes, Qualitative study

## Abstract

**Background:**

To explore patients’ experience of symptoms and impacts of Crohn’s disease (CD) and evaluate content validity of patient-reported outcome (PRO) measures in moderately-to-severely active CD.

**Methods:**

Qualitative, web-based interviews were conducted to elicit participants’ experiences with CD, and to obtain their feedback on: Patient Global Rating of Severity (PGRS:1 ‘none’ to 6 ‘very severe’), Urgency Numeric Rating Scale (Urgency NRS:0 ‘no urgency’ to 10 ‘worst possible urgency’), patient-reported symptoms from Crohn’s Disease Activity Index (CDAI; abdominal pain [AP] [severe, moderate, mild, none], well-being [WB] [terrible, very poor, poor, slightly under par, generally well], bowel movement [BM] count and stool frequency), and Bristol Stool Chart (type 1 ‘hard’ to type 7 ‘entirely liquid’), including understanding of response options, recall period and examination of meaningful improvement in CD symptoms.

**Results:**

Seventeen adults and 3 adolescents (mean age:37.7 years; 70% female) were interviewed. Overall, 41.2% of adults had a college degree or higher. Most participants reported experiencing fatigue (*n* = 19), bowel urgency (*n* = 18), diarrhea (*n* = 18), AP (*n* = 17), and/or stool frequency (*n* = 15). CD impacted participants’ daily activities (*n* = 20), mood/emotions (*n* = 19), social activities/relationships (*n* = 19), productivity/performance at work/school (*n* = 18), and/or sleep (*n* = 15). Participants found PRO items and response options easy to understand and use and reported that it was easy to recall their symptoms over 24 h. Participants considered a 1- or 2-point reduction on PGRS as meaningful improvement. Seventeen participants considered 3-point improvement on Urgency NRS as meaningful. Participants with mild/moderate AP (*n* = 11) and severe AP (*n* = 13) considered a 1- and 2-point reduction in CDAI-AP score as meaningful improvement. Ten participants reported a 2-point reduction in CDAI-WB score as meaningful improvement if they had ‘terrible’ WB. Generally, participants considered going from 5 to 2 BMs/day as meaningful improvement. Nineteen participants considered improvement to stool type 3 to 5 on Bristol Stool Chart as meaningful.

**Conclusions:**

Bowel urgency, diarrhea, and AP were the most common CD symptoms. Cognitive debriefing findings support content validity of PRO measures in patients with CD and provide insights on levels of change in PRO measures considered meaningful by patients.

**Supplementary Information:**

The online version contains supplementary material available at 10.1186/s41687-025-00959-1.

## Background

Crohn’s disease (CD) is a chronic inflammatory bowel disease (IBD) that primarily affects the gastrointestinal tract [1]. The estimated prevalence of CD in the United States (US) is greater than 1 million with the highest incidence reported among adolescents [2, 3].

Cardinal symptoms of CD include abdominal pain (AP), bowel urgency, diarrhea, weight loss, and fatigue, which typically tend to appear in individuals aged between 15 and 30 years [1, 2]. Additionally, CD significantly affects patients’ functionality, day-to-day tasks, overall well-being, and capacity to work, contributing to economic and health-related quality of life burden [4].

Diarrhea and AP in the CD population are routinely assessed in clinical trials and clinical practice [5–7]. Despite being highly prevalent and burdensome [8, 9], the assessment of bowel urgency is not incorporated in commonly used disease activity measures, like the Crohn’s Disease Activity Index (CDAI) [10]. This omission is often due to the challenges physicians face in fully understanding the impact of urgency, which can contribute to the psychosocial burden experienced by patients [11, 12].

The US Food and Drug Administration (FDA) has issued guidance to develop and use patient-reported outcome (PRO) measures to support claims in labeling for symptomatic endpoints [13]. Qualitative methods to establish content validity are crucial in ensuring that PRO measures are meaningful and relevant to the intended patients [14, 15]. Research evaluating the patient-perceived meaning and relevancy of PRO measures assessing key CD-related physical symptoms has been limited [10, 16].

This study explores the patient experience of CD and remission in CD by focusing on key CD symptoms, including AP, bowel urgency, and bowel movement (BM). Additionally, this study aims to cognitively debrief the Patient Global Rating of Severity (PGRS, single item PRO measure), Urgency Numeric Rating Scale (NRS), patient-reported symptoms from the CDAI, and Bristol Stool Chart, including response option interpretation and examination of meaningful improvement in CD symptoms. Thus, the current study uniquely contributes to the existing literature by highlighting specific thresholds of improvement and focusing on patient perspectives of meaningful change.

## Methods

### Study design and patient population

This was a noninterventional, cross-sectional, qualitative study conducted between May and July 2023 in the US. Adults and adolescents (aged ≥ 15 years) who met all of the following criteria were deemed eligible to participate in an interview: had a clinician-confirmed diagnosis of active CD for at least 6 months; self-reported having moderately-to-severely active CD; were receiving biologic and/or conventional therapy for the treatment of CD; and were able to read, speak, and understand English.

A recruitment vendor was responsible for recruiting, screening, and scheduling the interviews. The participants were identified by using the firm’s databases of pre-profiled adults and adolescents with CD, followed by a screening of the interested participants. Adults/adolescents who met the eligibility requirements were recruited to participate in the interviews.

Interviews were conducted among 20 adult and adolescent participants with CD to provide an adequate sample for information saturation (i.e., when no significant new information was identified by the interviewers), qualitative analyses, and assessment of the research objectives. Qualitative studies do not rely on power calculations to determine the appropriate sample size. The sample size of 20 participants is usually adequate to obtain a point of “informational redundancy” or “thematic saturation” in qualitative research exploring specific research objectives [17, 18].

Participants were also required to provide consent/assent to participate in the interview. For adolescents, their parents or legal guardians provided permission prior to the conduct of interview. Patients who had undergone any surgery for the treatment of CD and/or only received immunomodulator monotherapy (such as azathioprine and 6-mercaptopurine) for the treatment of their CD were excluded from this study.

### Interview procedures

Qualitative, web-based interviews, each scheduled for nearly 60 min, were conducted by 2 experienced interviewers. The interview started with a concept elicitation phase, followed by a cognitive debriefing phase. All interviews followed a semi-structured interview guide to ensure that data were collected systematically and consistently and that interview objectives were met while encouraging the spontaneity of responses and a conversational tone. Initially, caregivers were asked to join the web-based interview with their child. However, they could leave the conversation after giving permission for their child to participate, provided both the caregiver and the child were comfortable with their departure.

The methodology for conducting open-ended and cognitive patient interviews is in accordance with the FDA guidance on PRO measures to support claims in approved medical product labeling [13].

#### Concept elicitation phase

Interviewers asked general, open-ended questions aimed at encouraging participants to share their experiences with CD and its impact on their lives. Following this, participants were asked specific, targeted questions to ensure that key topics of interest were covered, even if they had not been mentioned spontaneously. These topics included symptoms related to stool frequency, AP, and bowel urgency, as well as their effects on daily life, such as social and psychological aspects. Interviewers also explored additional concepts that were important to the patients. Participants were asked questions about the symptoms they considered most bothersome and most important to treat.

#### Cognitive debriefing phase

Participants were asked to review and provide feedback on the PRO measures, including PGRS, Urgency NRS, patient-reported symptoms from CDAI, and Bristol Stool Chart and their corresponding response options. A detailed description of these PRO measures is provided in the following section. Specifically, participants were asked to “think aloud” as they responded to the items. Interviewers also posed follow-up questions to further elucidate participants’ question-answering process and their understanding of the items, recall periods, and response options and to evaluate whether the items were relevant to their experiences. Finally, using the item-response choices, participants were asked questions related to symptom remission and the amount of change necessary to be deemed meaningful from their perspective. For example, participants were asked “*You selected [respondent’s answer]. Using the scale provided*,* what amount of improvement would you consider meaningful? Tell me why*.” and “*If you experienced severe symptoms*,* using the scale provided*,* what amount of improvement would you consider meaningful? Tell me why*.” (Supplementary Table 1).

Participants were also asked to identify the response option on the PRO measures that they thought would indicate that a new treatment was successful.

### Patient-reported outcome measures

The Patient Global Rating of Severity (PGRS) is a single-item PRO measure (similar to Patient Global Impression of Severity [PGI-S]) developed to evaluate the severity of the patient’s overall CD symptoms during the past 24 h using a 6-point scale ranging from 1 (none) to 6 (very severe) [19]. The Urgency NRS is a content-valid PRO measure developed to assess the severity of the urgency over the past 24 h using an 11-point NRS ranging from 0 (no urgency) to 10 (worst possible urgency) [20]. The CDAI is a clinician-reported outcome measure that was developed to quantify the symptoms of patients with CD [21]. Out of the 8 components of CDAI, AP (range, 0 [no pain] to 3 [severe pain]; response options: severe, moderate, mild, none), general well-being (range, 0 [generally well] to 4 [terrible]; response options: terrible, very poor, poor, slightly under par, generally well), BM count (number of BMs), and stool frequency (number of very soft or liquid BMs) over the past 24 h were reviewed by participants in the present study. The Bristol Stool Chart is a widely used clinical outcome assessment that categorizes feces into 7 types (range, type 1 (hardest) to type 7 (entirely liquid); type 1 being ‘separate hard lumps’, type 2 being ‘sausage-shape but lumpy’, type 3 being ‘like a sausage but with cracks on surface’, type 4 being ‘like a sausage or snake, smooth and soft’, type 5 being ‘soft blob with clear-cut edges’, type 6 being ‘fluffy pieces with ragged edges, a mushy stool’ and type 7 being ‘watery, no solid pieces, entirely liquid’). This chart describes the shapes and types of stool and can be used to help characterize constipation and diarrhea [22].

### Analysis

Data were systematically collected from each interview using Excel-based field notes and audio files. After the interviews, all audio recordings were transcribed verbatim without identifiable information (e.g., names). The thematic analysis approach was used to analyze the results of the interviews, aided by field notes and interview transcripts [23]; such that dominant trends were identified in each interview and compared across the results of the other interviews to identify themes or patterns in the way that participants described their experiences with CD and their perspectives on what constitutes a meaningful change in symptoms or denote symptom remission using the PRO measures. Transcripts were coded by one primary analyst using a deductive presence/absence framework. A second analyst quality-checked all coding; disagreements were resolved by the project director. Saturation was assessed by conducting 20 interviews. No new codes or themes emerged in the final interviews. The data were descriptively summarized using number, number missing, frequency, percentage, mean, median, maximum/minimum range, and standard deviation.

### Ethical considerations

All study participants provided informed consent, including permission to publish their anonymized responses. This study was conducted in accordance with the ethical principles that have their origin in the Declaration of Helsinki and that are consistent with Good Pharmacoepidemiology Practices and applicable local laws and regulations, as appropriate. The Institutional Review Board at RTI-HS International reviewed this research. Participants received an honorarium for their participation.

## Results

### Patient population

The mean age of adult participants (*n* = 17) was 41.4 years, while adolescent participants (*n* = 3) had a mean age of 17 years. Most participants were females (adults: *n* = 12; adolescents: *n* = 2) and White (adults: *n* = 11; adolescents: *n* = 3). A majority of the participants were receiving either adalimumab (adults: *n* = 4; adolescents: *n* = 2) or infliximab (adults: *n* = 4; adolescents: *n* = 1) for CD treatment. Less than half of the adult participants (*n* = 7) had a college degree or higher. Thirteen adult participants self-reported moderate CD, and 2 adolescent participants reported severe CD at the time of recruitment (Table 1). The average time since CD diagnosis was 9.7 years (range: 0.5 to 29 years) among participants.

**Table 1:** Demographic and clinical characteristics of participants

Characteristic	Adults (*n* = 17)	Adolescents (*n* = 3)	Total (*N* = 20)
**Age**,** mean years (range)**	41.4 (21–60)	17 (—)	37.7 (17–60)
Sex assigned at birth, n (%)			
Female	12 (70.6)	2 (66.6)	14 (70.0)
Male	5 (29.4)	1 (33.3)	6 (30.0)
Race and ethnicity, n (%)			
Asian	1 (5.9)	0 (0.0)	1 (5.0)
Biracial	1 (5.9)	0 (0.0)	1 (5.0)
Black	3 (17.6)	0 (0.0)	3 (15.0)
Hispanic	1 (5.9)	0 (0.0)	1 (5.0)
White	11 (64.7)	3 (100)	14 (70.0)
Highest educational level, n (%)			
Technical or associate degree	5 (29.4)	—	5 (25.0)
Some college degree	5 (29.4)	—	5 (25.0)
College degree	3 (17.6)	—	3 (15.0)
Professional or advanced degree	4 (23.5)	—	4 (20.0)
Employment status, n (%)			
Full-time	10 (58.8)	—	10 (50.0)
Disability	3 (17.6)	—	3 (15.0)
Not employed/retired	3 (17.6)	—	3 (15.0)
Student	1 (5.9)	—	1 (5.0)
Time since diagnosis (years), n (%)			
0.5 to 4	3 (17.6)	1 (33.3)	4 (20.0)
5 to 10	7 (41.2)	2 (66.7)	9 (45.0)
11 to 20	6 (35.3)	0 (0.0)	6 (30.0)
More than 20	1 (5.9)	—	1 (5.0)
**Self-reported CD severity**,** n (%)**			
Moderate	13 (76.5)	0 (0.0)	13 (65.0)
Moderate-to-severe	1 (5.9)	1 (33.3)	2 (10.0)
Severe	3 (17.6)	2 (66.6)	5 (25.0)
Treatment for CD, n (%)			
Immunomodulators	1 (5.9)	0 (0.0)	1 (5.0)
Adalimumab	4 (23.5)	2 (66.7)	6 (30.0)
Certolizumab pegol	1 (5.9)	0 (0.0)	1 (5.0)
Infliximab	4 (23.5)	1 (33.3)	5 (25.0)
Ustekinumab	3 (17.6)	0 (0.0)	3 (15.0)
Risankizumab	2 (11.8)	0 (0.0)	2 (10.0)
Vedolizumab	4 (23.5)	0 (0.0)	4 (20.0)

CD = Crohn’s disease; n = number of participants; N = total number of participants

### Concept elicitation

#### Crohn’s disease symptoms

**Fig. 1:**
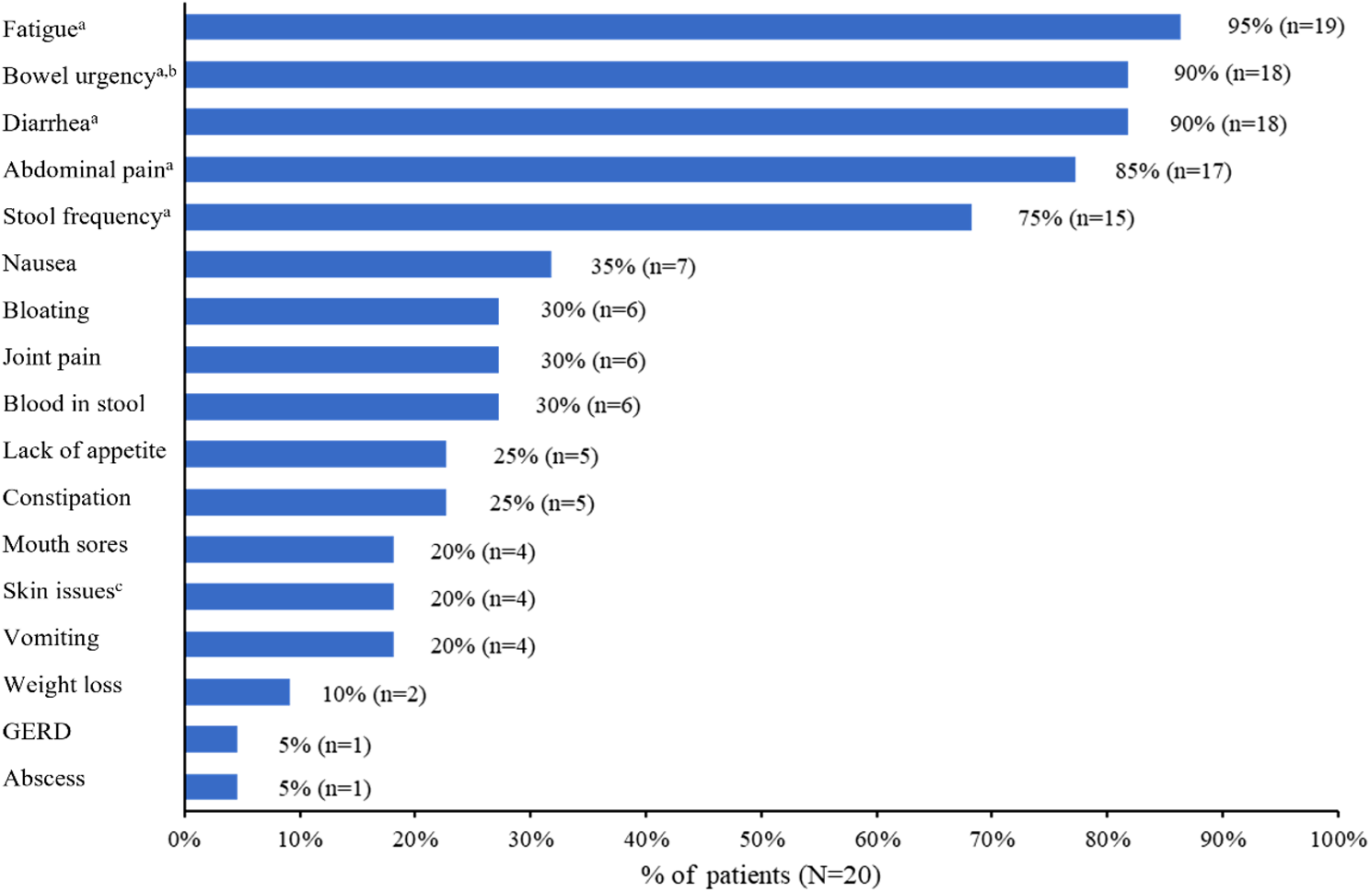
Proportion of participants who reported experiencing Crohn’s disease symptom. GERD = gastroesophageal reflux disease; n = number of participants; N = total number of participants. ^a^Symptoms were probed during the interview if not spontaneously reported by participants. ^b^Sudden or immediate need to pass stool. ^c^Skin issues included sores, rash, psoriasis, and contact dermatitis

Most participants reported experiencing fatigue (*n* = 19), bowel urgency (*n* = 18), diarrhea (*n* = 18), AP (*n* = 17), and/or stool frequency (*n* = 15; Fig. 1). In addition, 6 participants spontaneously reported experiencing joint pain, and 5 participants spontaneously reported experiencing constipation due to their CD.

#### Most bothersome and most important to treat Crohn’s disease symptoms

**Fig. 2:**
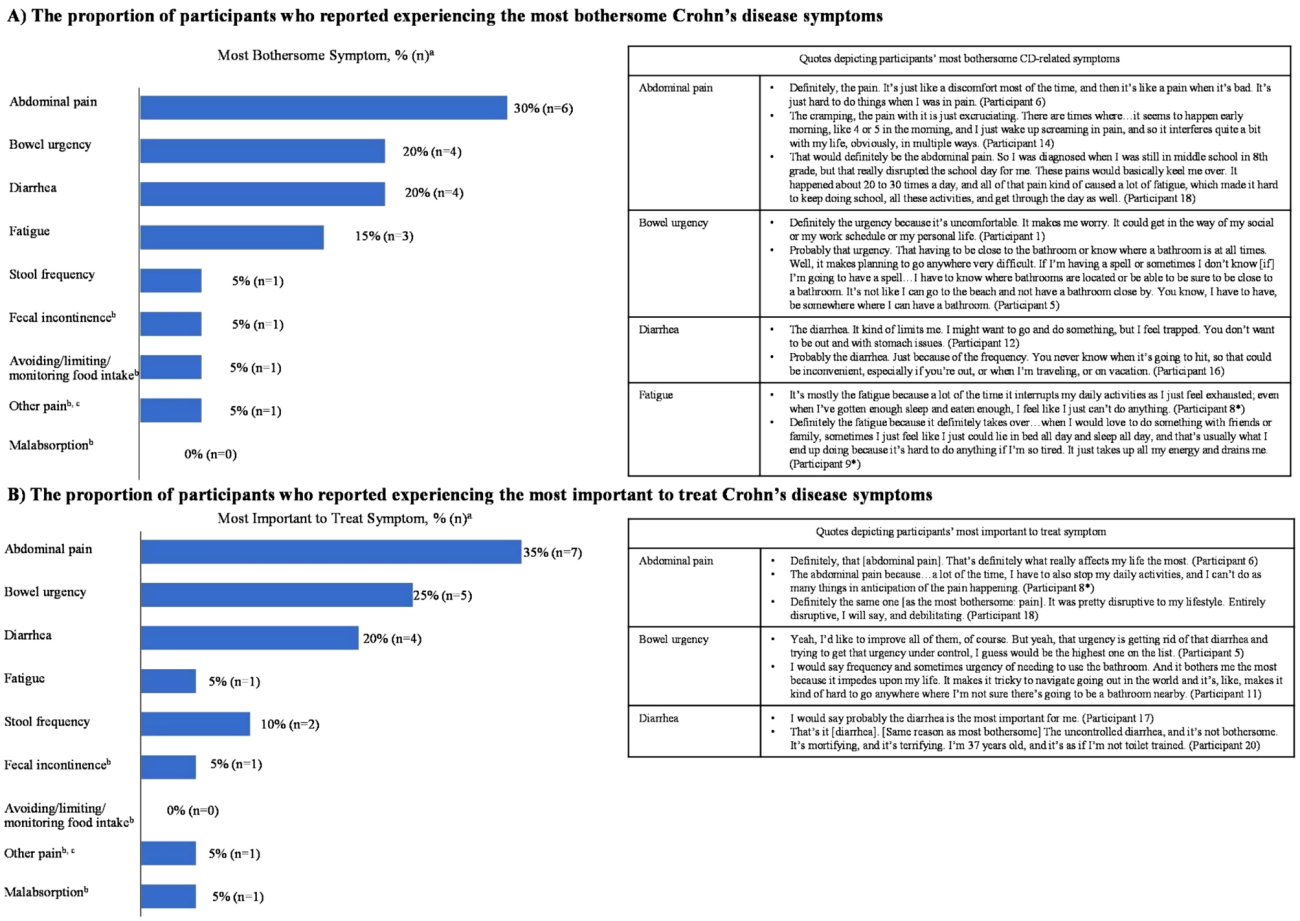
Most bothersome and most important to treat symptom . n = number of participants. ^a^Participants could select more than 1 symptom. ^b^Symptoms were not probed during the interview but were spontaneously reported by participants. ^c^Pain when passing stool. *Asterisk following the Participant number signifies adolescent participants

AP (*n* = 6), bowel urgency (*n* = 4), diarrhea (*n* = 4), and fatigue (*n* = 3) were the 4 most bothersome symptoms experienced and reported by participants. Stool frequency, fecal incontinence, avoiding/limiting/monitoring food intake, and other pains were experienced and reported by 1 participant each as the most bothersome symptom (Fig. 2A).

A similar response was observed when participants were asked for the most important CD symptom to treat. AP (*n* = 7, including 3 adolescent participants), bowel urgency (*n* = 5), and diarrhea (*n* = 4) were reported as the most important symptoms to treat (Fig. 2B).

#### Impact of Crohn’s disease on patients’ daily activities

**Fig. 3:**
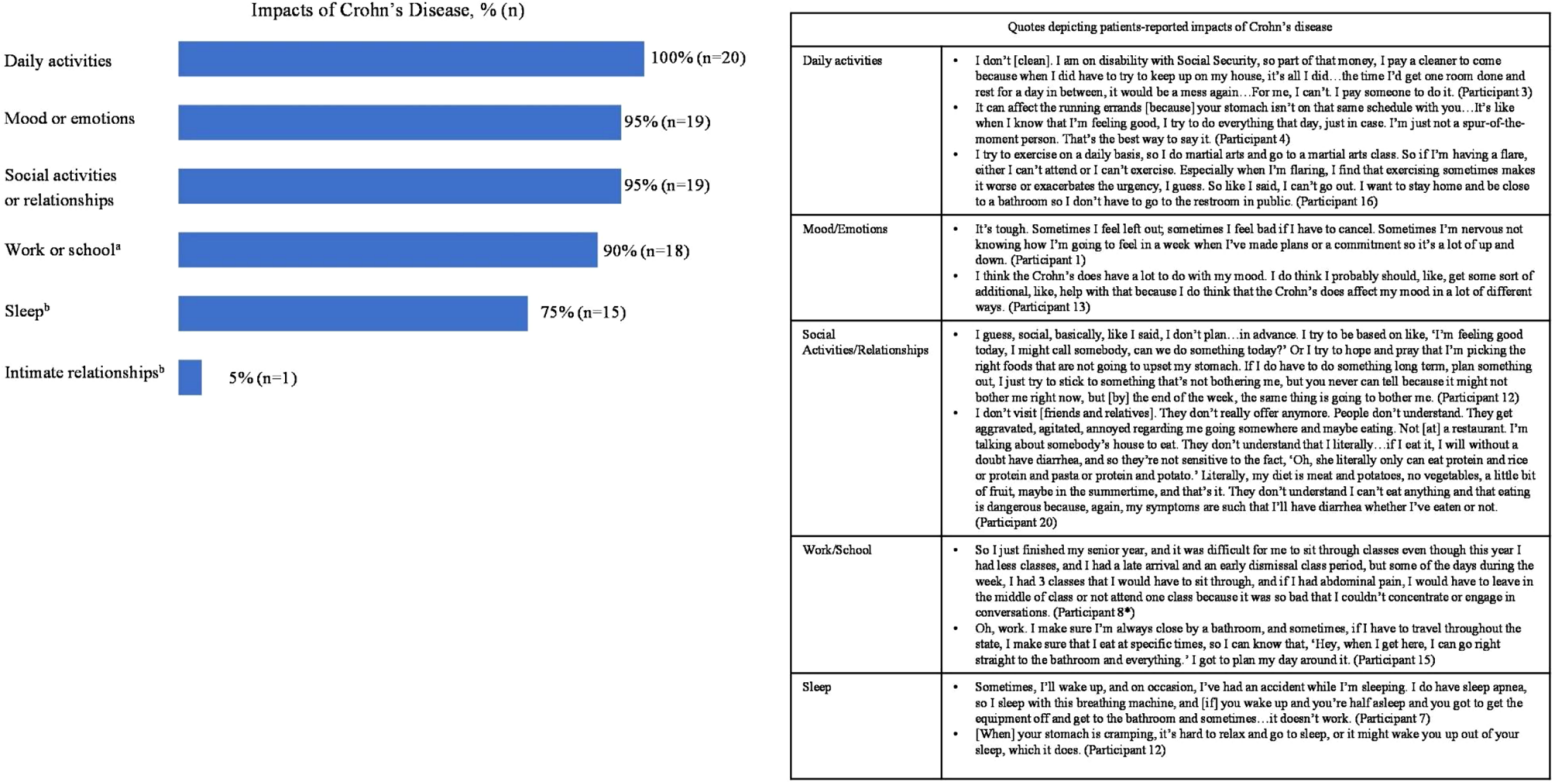
Participant-reported impacts of Crohn’s disease. ^a^Impact on work or school included loss of productivity, tardiness, and absenteeism. ^b^Impact was not systematically probed during the interview but was spontaneously reported by participants. *Asterisk following the Participant number signifies adolescent participants

All participants (*N* = 20) reported that CD impacted their daily activities, including cleaning, cooking, doing errands, caring for children, exercising, or playing sports. Nineteen participants reported that CD affects their mood or emotions (e.g., irritability, agitation, frustration, annoyance, embarrassment, anxiety, and sadness) and their social activities or relationships (*n* = 19; Fig. 3). Eighteen participants reported that CD has limited their ability to focus, thereby impacting performance and productivity at work or school. Fifteen participants reported that CD symptoms such as fatigue, pain, or bowel urgency interrupt and impair their sleep (Fig. 3).

### Cognitive debriefing

#### Patient global rating of severity

All participants (*N* = 20), including adolescents, could understand and respond to the PGRS, which asked, *“How would you rate your overall Crohn’s Disease symptoms over the past 24 hours?”* Additionally, they reported that recalling their overall CD symptoms over “the past 24 hours” was easy. They also found the response options (i.e., very severe, severe, moderate, mild, very mild, and none) straightforward, easy to select, and could interpret the meaning. Participant-reported interpretations of their responses are shown in Supplementary Table 2.

Participants generally reported that a 1- or 2-point reduction in score on the PGRS would be a meaningful improvement to them. For example, 6 participants reported if they were experiencing severe symptoms, a 1-point change in severity (i.e., moderate) would be a meaningful improvement. Conversely, 3 participants (including 1 adolescent) reported a 3-point reduction, and 1 participant reported that a 4-point reduction would be a meaningful improvement if they were experiencing severe symptoms (Table 2).

Participants interpreted “meaningful improvement” as a reduction in symptom severity that impacted their daily lives. Some participants described meaningful improvements in their condition, for example one noted: “*I would say to the mild [from severe]. I would say that [is] where I am now*,* [which] is definitely meaningful…because it doesn’t affect me on a daily basis. I didn’t really use to…have good days that [were] mild…so the fact that [the] majority of my time is fine*,* I don’t have to stop what I’m doing”* [Participant 6]. Another shared “*Just one down to mild [would] be very meaningful and nice. [I would have] more chances to go out and do things [and] not having to think about all sorts of challenges*,* knowing that I would be okay and not so worried about things*” [Participant 19].

**Table 2:** Changes in responses options considered a meaningful improvement on the patient global rating of severity

Starting point	1-point improvement	2-point improvement	3-point improvement	4-point improvement
Very severe	0	2	0	0
Severe	6	8	3	1
Moderate	3	6	0	0
Mild	1	2	0	0
Very mild	1	0	0	0
None	0	0	0	0
Total	11	18	3	1

The numbers reported in the table refer to the number of responses (not participants)

Most participants believed that to consider a treatment successful, their CD symptoms would either be mild (*n* = 9) or very mild (*n* = 6) on the PGRS (Table 3). Moreover, 18 participants reported that their overall CD symptoms would not need to be resolved entirely for them to consider a treatment successful. The remaining 2 participants, both adolescents, reported that their symptoms would need to be completely resolved to consider a treatment successful.

**Table 3:** Participant-reported clinical outcome assessments response for new treatment to be considered successful

Response option	*n* (%)	Quotes specified by participant
Patient Global Rating of Severity, *N* = 20 “How would you rate your overall Crohn’s Disease symptoms over the past 24 h?”		
Moderate	2 (10.0)	[Participant 13] “*I would say moderate just because it’s like an everyday*,* it is just part of my life*,* and…the urgency is like unpredictable*,* and it’s like sometimes there*,* and it’s sometimes not.”*
Mild	9 (45.0)	[Participant 8, adolescent] “*When my symptoms are mild*,* I’ve noticed that…very quickly*,* even the next day*,* have moderate symptoms*,* and when I have periods that I have no symptoms*,* I’m more likely to stay in that period for longer*,* and that’s when I feel like I did before I had symptoms…and that’s when I consider my quality of life to be much better as I’m not anticipating something getting worse the next day*,* and I’m not really planning things around anticipated symptoms.”*
Very mild	6 (30.0)	[Participant 12] “*I guess very mild. I’m still thinking that that would just be borderline what a normal person would go through. You might get an upset stomach*,* but it’s nothing like I’m used to. It’s just something that happens sometimes. You might eat the wrong thing or [have a] virus*,* not just everyday issue that might flare up.”*
None	3 (15.0)	[Participant 4] “*I would say none since this is something new*,* hoping that it would just stop it altogether*,* the symptoms.”*
Urgency Numeric Rating Scale, *N* = 18 “How severe was your urgency (sudden or immediate need) to have a bowel movement in the past 24 h?”		
1	3 (16.7)	[Participant 8, adolescent] A 1. “*1 would be*,* I might experience urgency*,* but I would not be able to distinguish it from just normal bathroom urgency as in there would be no abdominal pain accompanied with it*,* and I wouldn’t be able to distinguish that it’s from my Crohn’s disease.”*
2	5 (27.8)	[Participant 17] “*I would want to be at a 2. I would say not very urgent. To me…it wouldn’t be a 0*,* of course*,* no urgency*,* but again being able to wait till I found a bathroom or going home*,* or I could still function going to the store and stuff like that and wait till I got home to go to the bathroom.”*
3	7 (38.9)	[Participant 12] “*If I get to a 3. I think…my body would signal to me that I did have to use the bathroom*,* but I would be able to hold it and get to my destination if I was driving and not get a ticket or finish what I’m doing…without rushing down the hallway or anything.”*
4*	1 (5.6)	
5	2 (11.1)	[Participant 7] A 5. “*I’m currently on a medication*,* and I am significantly better than I was*,* but I still feel terrible.”*
The Crohn’s Disease Activity Index		
Abdominal pain, *N* = 20 “Please think about your worst pain experience in the past 24 h. How would you rate your abdominal pain over the past 24 h?”		
Moderate	1 (5.0)	[Participant 3] “*I think going from severe to moderate would be a success…because that’s gaining improvement. A success would go [from] severe to mild because you are actually managing it more*,* and it’s proven to be working.”*
Mild	11 (55.0)	[Participant 13] “*I think that ideally mild*,* but if I was severe at a large percent of the time*,* and I went to moderate*,* I think that would still be successful in my book.”* [Participant 17] “*I would say*,* ‘Mild.’ I would say…just going from being in so much pain and having it affect my joints and all that stuff and being able to eat again and taking some medicine*,* that would be good.”*
None	8 (40.0)	[Participant 8, adolescent] “*I would consider severe to none to be successful…because for me*,* my symptoms can progress very quickly*,* and when I have mild pain*,* I’m usually anticipating something to get worse…but if I have no abdominal pain*,* it usually stays in that period for at least a couple of days or weeks. So*,* a severe to none reduction severely increases my quality of life*,* and that’s very meaningful to me.”*
Well-being, *N* = 20 “How would you rate your general well-being over the past 24 h?”		
Generally well	12 (60.0)	[Participant 3] “*Generally well or slightly under par. I think slightly under par would be more realistic…it would probably be the best that I could get. It would be able to push through your days and get the things done that you need to do. You can participate in life*,* but you’re still having that fatigue*,* and you still need to be aware of where the bathrooms are*,* but maybe not have to use them. [And would you consider a treatment successful if it was poor? ] I think I would because…my medications that I’m on now*,* they work enough to get me to poor and then the medications I have to supplement with help me to get to slightly under par*,* so if it’s a single medication getting you to poor*,* I would consider it a success.”* [Participant 10, adolescent] “*Generally well…because that’s how I feel like normal life should be. Most of the time you’re not feeling like you’re always sick. You can some days forget that you are*,* that you do have a disease that’s always with you*,* but just being able to live normally.”*
Slightly under par	6 (30.0)	[Participant 7] “*Slightly under par. I guess I’ve been sick for so long*,* that I don’t ever expect to feel good. Which is*,* I’m realizing as I’m answering these questions*,* I’m just aiming for better.”*
Poor*	2 (10.0)	
Bowel movement count, *N* = 20 “Please count each toilet visit during which you passed any amount of stool. Even if little time had passed since you last left the toilet, if you returned and passed stool again, please count this as a separate bowel movement. If you passed stool before making it to the toilet (had an accident), please count this as a bowel movement. How many bowel movements have you had in the past 24 h?”		
1	5 (25.0)	[Participant 10, adolescent] “*Also a 1. Just seems like a normal amount for me. Just based on previous experiences*,* that’s where I would want to be.”*
2*	7 (35.0)	
3*	2 (10.0)	
4	2 (10.0)	[Participant 16] “*Four*,* because it’s closer to an average or what a healthy person would have. So*,* it’s just a little above average versus severe.”*
5	2 (10.0)	[Participant 7] “*Five*,* because I know that I’m going to the bathroom way too much. It’s crazy to think it’s not realistic for my body to be able to go only 5 times.”*
6*	1 (5.0)	
7*	1 (5.0)	
The Bristol Stool Chart, *N* = 19		
Type 3	4 (21.1)	[Participant 15] “*Type 3 is what I would want to have that would be successful probably because the pain is more substantial when I have a type 4. I don’t know really [know] what I should be looking for or what is the best for your body*,* but type 3 is what I have when I’m not having*,* like I said*,* as many of the symptoms*,* so there’s less pain and that’s what I would want to go with.”*
Type 4	11 (57.9)	[Participant 18] “*I would pick type 4 probably. It seems to be a normal stool type*,* solid*,* passed easily and not either hard to pass and hard or entirely liquid.”*
Type 5	4 (21.1)	[Participant 17] “*I would say type 5. It would be a step up from the liquid*,* and it would be better than what I had before. From number 7 to number 5 would be definitely an improvement.”*

*Verbatim patient responses were unavailable for a few participants

#### Urgency numeric rating scale

All participants (*N* = 20) reported that they were able to understand and respond to the Urgency NRS. Participants who had only 1 BM or 2 or more BMs with similar levels of bowel urgency in the past 24 h interpreted and reported the response options consistently. Among the participants who had more frequent BMs with varying degrees of bowel urgency, some participants reported experiencing average bowel urgency in the past 24 h, and some participants reported experiencing their worst bowel urgency in the past 24 h. All participants (*N* = 20) found the Urgency NRS straightforward and easy to select a response; however, 5 participants reported that fewer options (a scale of 0 to 5) would make it easier to select a response compared with a 0 to 10 scale. The quotes used by participants indicated that they could interpret the meaning of the response options on the Urgency NRS (Supplementary Table 2).

Most participants would consider a new treatment successful if their urgency corresponded to a 2 (*n* = 5) or 3 (*n* = 7) on the Urgency NRS. Three participants believed that an urgency score of 1 would be needed to consider a new treatment successful (Table 3). Nineteen participants reported that their bowel urgency would not need to be entirely resolved to consider a treatment successful. One participant was not asked this question because they reported not experiencing bowel urgency due to their CD. From the reported bowel urgency, a 3-point improvement was considered a meaningful improvement among participants (*n* = 17) (Table 4).

**Table 4:** Amount of improvement on the urgency numeric rating scale that was meaningful to participants

Amount of improvement	Improvement from reported urgency (*n* = 17)
0 points	3 (17.6)
1 point	0 (0.0)
2 points	2 (11.8)
3 points	4 (23.5)
4 points	2 (11.8)
5 points	4 (23.5)
6 points	1 (5.9)
7 points	0 (0.0)
8 points	1 (5.9)
Mean (SD)	3.4 (2.2)
Median	3

SD = standard deviation

#### Crohn’s disease activity index-abdominal pain

Overall, participants were able to easily provide a response to the CDAI-AP item, considering “worst pain” as the worst AP. However, 3 participants found the instructional text confusing and were unsure whether to compare their AP to their worst pain (e.g., a migraine) or if they should rate their worst AP. Thus, they suggested that “abdominal” should be added to the instructional text for clarity. All participants (*N* = 20) found the response options (i.e., severe, moderate, mild, and none) clear and reported that it was easy to recall their worst AP over the past 24 h ([B] Supplementary Table 2).

Of the 20 participants, 7 reported experiencing moderate, 6 reported experiencing mild, and 7 reported experiencing no AP. Of those who reported experiencing mild or moderate AP (*n* = 13), 11 reported a 1-point reduction in score as a meaningful improvement. If they were experiencing severe AP, a 2-point reduction (i.e., mild) and a 1-point reduction (i.e., moderate) in score would be considered a meaningful improvement by 13 and 6 participants, respectively. Most participants reported that improvement to mild (*n* = 11) or no AP (*n* = 8) would be needed to consider the treatment successful (Table 3). A total of 15 out of 19 participants reported that their AP would not need to be resolved entirely for them to consider a treatment successful. On the contrary, 4 participants (including 2 adolescent participants) reported that their AP would need to be resolved entirely for them to consider a treatment successful.

#### Crohn’s disease activity index-well-being

All the participants understood and responded to the CDAI well-being item. Except for 1 participant, the remaining 19 interpreted the item consistently and reported their well-being in terms of their CD. That 1 participant reported *“A lot of things contribute to my well-being*,* and sometimes it’s hard to know if it was my [CD-related] symptoms…affecting my well-being [or] just other life things affecting my well-being…but when I answered it*,* I was attributing it to how my Crohn’s symptoms affected my well-being.”* Participants found the response options (i.e., terrible, very poor, poor, slightly under par, and generally well) straightforward and easy to select a response. They also reported that it would be easy for them to recall their general well-being over a 24-hour period.

At the time of the interviews, 17 out of 20 participants reported their general well-being as “generally well” (*n* = 8) or “slightly under par” (*n* = 9). The participants who reported “poor” (*n* = 2) or “very poor” (*n* = 1) well-being considered a 1-point reduction in score to be a meaningful improvement given their current well-being. Ten participants reported that a 2-point reduction (i.e., poor) would be a meaningful improvement if they were experiencing “terrible” well-being. Additionally, 8 participants considered a 3-point reduction (slightly under par) meaningful, and 2 participants considered a 4-point reduction (generally well) as a meaningful improvement. Twelve participants reported that their general well-being would have to be “generally well” to consider a new treatment successful (Table 3). Of these, 9 participants reported they would still consider the treatment successful if their general well-being was “slightly under par.”

#### Crohn’s disease activity index-bowel movement count

Most participants found the instructional text of the CDAI-BM count item clear and easy to understand (*n* = 18); however, 2 participants suggested the following edits for clarity: [Participant 18] *“The only slight thing that came to mind was the first sentence says*,* ‘Please count each toilet visit…’ but later on*,* it says*,* ‘If you pass stool before making it to the toilet or had an accident*,*’ to also count that*,* so it’s a little contradictory. I would say just taking out that ‘toilet visit’ language and just saying*,* ‘Please count each time that you pass any amount of stool’ would clarify that.”* and [Participant 16] *“It’s wordy. [But is anything confusing? ] Yeah*,* the second sentence. I think you could simplify that…to say*,* ‘If you have a BM and needed to return to pass stool again*,* count this as a separate BM.’ I think it could be edited down and clarified for simplicity.”*

Participants were able to easily understand and provide a response to the CDAI-BM count item and reported that it would be easy for them to recall how many BMs they had over a 24-hour period.

Participants reported that they would generally consider going from having approximately 5 BMs to 2 BMs per day as a meaningful improvement. Overall, most participants reported that if they had 1 (*n* = 5) or 2 (*n* = 7) BMs daily, they would consider a new treatment successful (Table 3). A mean of 2.8 daily BM was reported as the desired BM count with a successful treatment.

#### Crohn’s disease activity index-stool frequency

All the participants (*N* = 20) could understand and respond to the CDAI stool frequency item. Participants could recall the number of very soft or liquid BMs they had in the past 24 h, which varied from 0 to 18; thus, the number of BMs considered as meaningful improvement was wide-ranging. In contrast to reducing the number of very soft or liquid BMs, 2 participants spontaneously mentioned that reducing the number of BMs or improving bowel urgency was more important to them. Eighteen participants reported that they would consider a new treatment successful if they had, on average, 2 very soft or liquid BMs daily. Of these, 12 participants reported that complete resolution of this symptom was not needed for them to consider the treatment successful.

#### Bristol stool chart

Most participants (*n* = 19) found the text in the Bristol Stool Chart easy to understand, and it was easy for them to recall the type of BMs in the past 24 h. However, 1 out of 19 participants recommended that it would be easier if the examples pictorializing the different types of stools were a bit larger to discern the different types. One adolescent participant reported that it was harder to recall the type of BMs unless it was on the far end of the spectrum as they do not pay attention to it.

A total of 19 participants reported that an improvement to stool type 3 to 5 would be needed for them to consider the treatment successful (Table 3).

## Discussion

The growing appreciation for patient perspectives on their disease burden and improvement has placed high importance on using reliable and content valid PRO measures in clinical trials [14]. To capture the experiences and dimensions of CD that are important to patients, this qualitative study included concept elicitation that focused on soliciting patients’ symptoms and their impacts, followed by cognitive debriefings of PGRS, Urgency NRS, patient-reported components of CDAI, and Bristol Stool Chart.

There is no known cure for CD, and the goals of management are symptomatic and endoscopic remission [24–26]. As patients with CD identify bowel and systemic symptoms as key physical symptoms, often reflecting disease activity [27], there is a need to consistently assess and report these symptoms using reliable PRO measures.

During the concept elicitation phase, most participants reported experiencing fatigue, bowel urgency, diarrhea, AP, and/or stool frequency. Previous qualitative studies also documented AP, diarrhea, bowel urgency, fatigue, and stool frequency as the most common physical symptoms among patients with CD in the US [11, 28, 29]. Rubin et al. also noted that patients perceived bowel urgency as one of the most bothersome CD symptoms [11]. Our study findings suggest that patients frequently describe bowel urgency a critical symptom impacting their daily lives, independent of stool frequency and consistency. Thus, recognizing bowel urgency as an independent symptom is crucial for developing comprehensive PRO measures that accurately capture the patient’s experience. Moreover, while patients reported experiencing a high impact of bowel urgency on quality of life, physicians rated bowel urgency as a low-impact symptom [11]. Likewise, another qualitative study also reported that bowel urgency symptoms impact their daily activity, health-related quality of life, and emotional well-being [30]. Consistent with these studies, we also found that bowel urgency is one of the most prevalent, bothersome, and important to treat symptoms, among patients with moderately-to-severely active CD.

All study participants reported that CD affects their daily activities. Most participants reported that CD impacted their mood or emotions; social activities or relationships; and productivity at work/school. In addition, several participants spontaneously reported that CD affected their sleep. In a previous study, approximately 50% of patients reported that CD had a high impact on their emotional mood [11].

Several previous studies have examined the relevance and comprehensiveness of the Urgency NRS, the CDAI, and the Bristol Stool Chart individually in different populations (i.e., healthy adults and patients with ulcerative colitis [UC] or IBD) [10, 16, 31]. The present study simultaneously also examined the content validity of these PRO measures in the CD population. The cognitive debriefing results indicated that despite the diverse educational backgrounds, most patients understood and easily completed the PGRS, the Urgency NRS, the patient-reported symptoms of CDAI, and the Bristol Stool Chart. Moreover, the content validity of the Urgency NRS was well-demonstrated among patients with UC [32], which corroborates with our findings in patients with CD.

Participants’ spontaneous narratives about their symptoms closely aligned with their responses to the PRO items. For example, many participants mentioned AP, bowel urgency, and diarrhea as their most bothersome symptoms, which were also the primary focus of the PRO measures. Furthermore, the verbatim accounts from participants underscore the impact of these symptoms on their daily lives, highlighting that improvements in these symptoms would enhance their overall well-being. This alignment reinforces the content validity of the PRO measures utilized in our study. Study participants were able to easily identify which response options on each of the PRO measures would correspond to a meaningful improvement from the current severity of their symptoms and if a new treatment was successful. Based on their experiences, all adult participants reported that their CD-related symptoms, including bowel urgency, diarrhea, and AP, would not need to be completely resolved for them to consider a treatment successful. On the contrary, 2 out of the 3 adolescents, who reported that their overall CD symptoms were “mild,” reported that they would consider a treatment successful if their symptoms were completely resolved.

Consistent assessment of CD symptoms and understanding their impact on patients might allow informed allocation of healthcare resources and identify the unmet treatment needs, resulting in improved patient outcomes. Moreover, considering patients’ perspectives and their expectations while making treatment decisions are crucial for the treatment success [33].

### Limitations

Though saturation was reached on the total sample size, only 3 adolescents were included in this study. Therefore, content validity for adolescents cannot be confirmed and should be interpreted with caution. Moderately-to-severely active CD was defined based on participants’ self-perception of CD, as opposed to clinician-confirmed disease activity or severity. However, the clinician-confirmed diagnosis, treatment use, and time since CD diagnosis were also considered and support the self-reported CD severity of study participants. The racial diversity was limited within the recruited population which could impact the generalizability of the findings to some racial subgroups. A psychometric evaluation of PRO measures is planned to explore their validity, reliability, and further estimate interpretations of meaningful change among patients with CD.

## Conclusions

In summary, the concept elicitation data suggested that AP, bowel urgency, and diarrhea were the most bothersome and important to treat symptoms for patients with CD. Findings from the cognitive debriefing supported the content validity of the PGRS, Urgency NRS, CDAI, and Bristol Stool Chart among patients with CD. Additionally, these results demonstrated that despite different educational backgrounds, participants were able to understand and easily complete the PRO measures. This research also provided qualitative evidence on the amount of change that would be meaningful to participants, which provides additional supportive evidence for future quantitative analyses conducted using clinical trial datasets and real-world evidence studies.

## Electronic supplementary material

Below is the link to the electronic supplementary material.

Supplementary Material 1

## Data Availability

The datasets generated and/or analyzed during the current study are not publicly available due to privacy and ethical concerns.

## Abbreviations

AP: Abdominal pain
BM: Bowel movement
CD: Crohn’s disease
CDAI: Crohn’s Disease Activity Index
FDA: Food and Drug Administration
IBD: Inflammatory bowel disease
NRS: Numeric rating scale
PGRS: Patient Global Rating of Severity
PRO: Patient-reported outcome
RTI-HS: RTI Health Solutions
SD: Standard deviation
UC: Ulcerative colitis
US: United States of America
WB: Well-being
