# Supplementary material for: Exploring patient-reported outcome measures to assess symptoms of moderately-to-severely active Crohn’s disease in adult and adolescent patients: a qualitative study
Source: J Patient Rep Outcomes. 2025 Nov 4;9:128. doi: 10.1186/s41687-025-00959-1 (PMC12586775; doi:10.1186/s41687-025-00959-1)
Supplement: Supplementary file 1 — Supplementary Material 1 [file 41687_2025_959_MOESM1_ESM.docx]

SUPPLEMENTARY MATERIAL

1. Supplementary Table 1. Sample Questions and Probes from Interview Guide

| Question | Scripted Probes |
| --- | --- |
| **Patient Global Rating of Severity** | - You selected [respondent’s answer]. Using the scale provided, what amount of improvement would you consider meaningful? Tell me why.* - If you experienced severe symptoms, using the scale provided, what amount of improvement would you consider meaningful? Tell me why.* - [If not mentioned.] In terms of your Crohn’s symptoms, what would you consider a treatment success? |
| **Urgency Numeric Rating Scale** | - [If not mentioned.] In terms of your urgency, what would you consider a treatment success? - [If not mentioned.] Would your bowel urgency need to be completely resolved for you to consider a treatment successful? Why or why not? If yes, how long would your symptoms need to be completely resolved? |
| **Crohn's Disease Activity Index (CDAI) Abdominal Pain and Well-Being** | - [If not mentioned.] In terms of your abdominal pain, what would you consider a treatment success? - [If not mentioned.] Would your abdominal pain need to be completely resolved for you to consider a treatment successful? Why or why not? If yes, how long would your symptoms need to be completely resolved? - If you experienced terrible well-being, using the scale provided, what amount of improvement would you consider meaningful? Tell me why. |
| **Bowel Movement Count** | - [If not mentioned.] In terms of bowel frequency, what would you consider a treatment success? - [If not mentioned.] Would the frequency need to be completely resolved for you to consider a treatment successful? Why or why not? If yes, how long would your symptoms need to be completed resolved? |
| **CDAI** | - [If not mentioned.] In terms of how many bowel movements you have that are liquid or very soft, what would you consider a treatment success? |
| **Bristol Stool Chart** | - [If not mentioned.] If you were to have bowel movements that were types 3 or 4 on the chart, would you consider a treatment successful? Why or why not? |

*Questions common for Patient Global Rating of Severity, Urgency Numeric Rating Scale and CDAI Abdominal Pain

**(B) Supplementary Table 2. Participant-Reported Interpretation of Response Options**

| **Patient Global Rating of Severity** |
| --- |
| - [Participant 15] *Very mild. What it is, basically it’s almost none, but I can still…my stomach is still disruptive, and I can feel it, but it’s not a pain [to deal with].* - [Participant 9, adolescent] *I would say mild. I’ve been dealing with some stomach pain, [and] I have had a couple of urgent bathroom stops, but other than that, I would just say it’s been constant…* - [Participant 5] *I would say right now it’s kind of in moderate. I’m having some diarrhea, but it hadn’t been real bad. The cramping hadn’t been real bad. It is just a little bit. Not real severe, not really having to dash to the bathroom. When I go to the bathroom, I’ll have a little bit of diarrhea.* - [Participant 20] *They have been very severe…I had an episode today. I got to the toilet, but I made a mess of the toilet at the doctor’s office. Yesterday, I had 2 very explosive diarrhea bowel movements, and I hadn’t even eaten anything, which led me to have nausea.* |
| **Urgency Numeric Rating Scale** |
| - [Participant 18] *I would say 0. I’d say no urgency…while there’s times that I feel the need to use the bathroom, it’s definitely not an urgency. An 8 would start to be pretty sudden and immediate. I would say that probably kickstarts a bit of a hurry to get to the bathroom [but] not an all-out rush or panic, [which would be] a 9 or a 10, maybe. [What would a 2 look like?] I would consider that little to no urgency. I would say, you feel the need to go to the bathroom, and then at some point you’re like, ‘I got to go now,’ so you just kind of at your leisure, go and use the bathroom.* - [Participant 8] *I would answer a 2, and that’s because last night I did have a little bit of urgency, but it was not bad, where I needed to find a bathroom immediately. It was just a little more than I would normally feel.* - [Participant 16] *I would say 5. Most of the time yesterday I didn’t have urgency. One time yesterday I had some urgency, but it wasn’t severe urgency. It wasn’t an emergency. It was more just a rush to get to the bathroom, but nothing where I was worried about having an accident.* - [Participant 13] *Let’s say an 8. Having to race to the bathroom and like, I guess kind of like, just dropping whatever I’m like doing to go to the bathroom.* - [Participant 7] *It’s at a 9. The last few weeks have been pretty rough, and I just have been running to the bathroom constantly. [What’s the lowest you would ever score on this scale?] Maybe a 5, [which] means I can go on a long car trip, well, like a half hour car trip, and not have to stop to go to the bathroom.* |
| **Crohn’s Disease Activity Index-Abdominal pain** |
| - [Participant 9, adolescent] *I would say, ‘None.’ Yeah, there was no pain.* - [Participant 13] *I would say, ‘Mild.’ In the past 24 hours, I haven’t had that much abdominal pain. When I was using the restroom, there was a tiny bit, but I’ve had much, much worse. It wouldn’t come anywhere close to moderate or severe, certainly over the past 24 hours.* - [Participant 2] *Moderate. Within the last 24 hours I’ve been dealing with a lot of bloating that causes pain. It’s just there, and it’s causing the pain of feeling gassy and feeling I have to go.* - [Participant 19] *Moderate. I’m not in crazy pain. I’m fine, but I do have substantial pain, and it’s not mild. It’s not like a sore muscle, which I would say mild is like a sore muscle. Moderate is like, ‘Oh, this is a little bit of pain,’ and it’s really annoying, but it’s not severe.* |
| **Crohn’s Disease Activity Index-Well-Being** |
| - [Participant 3] *For me, that’s hard because yesterday was a hard day. I’m going to go with poor because yesterday was hard, but today’s better.* - [Participant 5] *I would say probably slightly under par. [Can you tell me why?] Well, I’m having the little bit of diarrhea, so I’m not feeling my best, so I guess I’m slightly under par.* - [Participant 15] *Okay. I would just say generally well. I’m not in any type of pain. I don’t have anything going on. I’m able to function. Okay today* - [Participant 20] *I would say very poor only because I wouldn’t say terrible because I have fortitude. Even though I feel terrible, I have to push through. I’m a grown adult with bills to pay. Yesterday, I took a 2 1/2-hour nap because I was so tired from all of the diarrhea, but there are days, like on Monday, I felt terrible literally. I had such stomach pain. I was balled over. I could not move.* |
